# Southern Dental Association

**Published:** 1870-07

**Authors:** S. J. Cobb


### ARTICLE III.

### *Southern Dental Association.*

Nashville, Tenn., May 10th 1870.

To the Editor of the *American Journal of Dental Science:*

Dear Sir:—Having just returned from New Orleans, where I have been attending a meeting of the Southern Dental Association, I propose a few lines for your Journal.

The meeting commenced on Wednesday morning, April the 15th, and continued until Saturday afternoon, making about four days. During this time we had a good deal of discussion, a majority present entering into it with that sort of generous and liberal spirit that always brings about a harmonious and pleasant state of affairs.

Notwithstanding this was the first regular meeting after organizing, it was a very good one, in fact a perfect success. It is true a few of the Southern States were not represented, which we hope will not be the case again; but while that was so. I am glad to say we had a good many Northern brethren with us, whom we were very glad to see. In future we hope to see many more, all are invited. The word Southern does not mean sectional, it just simply means a name for the Association. I am sure all present, and especially those from a distance were not only interested and pleased with the meeting, but delighted with the reception and treatment of the New Orleans Dentists. If ought should be said of them, it should be of their generous, liberal expenditure of means during our stay with them. The next meeting will be held in Charleston, S. C., commencing on the second Tuesday in April, 1871, at which time and place we hope to see not only every Southern State well represented, but a goodly number of the Northern. Having promised at the last meeting of the American Dental Association, to do all in my power to interest the profession South in the next meeting, which will be held in this place (Nashville, Tenn.) commencing on Tuesday Aug. the 2d.

I will say so far, I have left nothing undone, that I have been able to do, and will continue my efforts up to the day of the meeting.

From encouragement received from various parties South with whom I have been corresponding since my return from Saratoga last summer, and at New Orleans during the meeting of the Southern Association, I have reason to believe the South will be pretty well represented, and from what I know and hear of the North, I am of the opinion that the next meeting will be the largest we have ever had. As one of the committee of arrangements, I will say we are about ready for the meeting, having done the most of our work up to this time. We have managed to obtain a most excellent hall for the occasion. At a request, our Legislature passed a resolution granting us the privilege of using one of their halls. We have also seen the proprietors of the principal hotels in the city, and they have agreed to make liberal deductions to delegates who may stop with them. I have not heard from the transportation committee, but I have no doubt they will be able to get parties to and from Nashville for half price. It was done by our Southern Dental Association at New Orleans, without any trouble.

Yours Respectfully, S. J. Cobb.

---

### ARTICLE IV.

### *Wasted Alveolar Processes and Gums.*

By W. H. Atkinson, D.D.S., M.D.

**Read before the Southern Dental Association, April 14th, 1870.**

